# Analysis of single-cell RNA sequencing data to examine the gastric inflammation-to-cancer transition and evaluation of the effect of probiotic on precancerous lesions

**DOI:** 10.1016/j.engmic.2025.100208

**Published:** 2025-05-09

**Authors:** Minmin Hu, Shiyang Xu, Ruofei Xu, Xiangjie Qi, Xiaofeng Yu, Jinqi Wang, Yige Li, Yangyang Liu, Guiran Xi, Junbao Yu, Mei Shi

**Affiliations:** a State Key Laboratory of Microbial Technology, Institute of Microbial Technology, Shandong University, Qingdao 266237, China; b Department of Urology, Zibo Municipal Hospital, Zibo 255400, China

**Keywords:** Gastric cancer, Inflammation-to-cancer transition, Single-cell RNA sequencing, Probiotic

## Abstract

Gastric cancer (GC) is the fifth most prevalent malignancy globally. However, its heterogeneity and asymptomatic early-stage development hinder timely diagnosis and effective treatment. Here, we employed single-cell RNA sequencing to delineate the transitional features of pit mucous cells (PMCs) during the gastritis-to-cancer transition and identified 100 core genes. Characterization of the gene set revealed the role of ribosomal protein small subunit and ribosomal protein large subunit in inflammation-to-cancer transition, which promoted ribonucleoprotein complex biogenesis and cytoplasmic translation. External validation using independent cohorts confirmed that this core gene set discriminated disease progression (AUC > 0.7) and was significantly enriched in GC tissues (*p* < 0.01). Moreover, we evaluated the therapeutic intervention effects of *C. butyricum* and synbiotics (Weichanghao®) using a rat model of gastritis and demonstrated the targeted suppression of inflammation-to-cancer transition genes. Our findings establish the basis for early diagnosis of GC through PMC-driven molecular dynamics. Additionally, we propose microbiota-based strategies to prevent the inflammation-to-cancer transition in preneoplastic stages. Furthermore, our results highlight that dysbiosis of the gastric microbiome can be addressed using probiotic supplementations and the core gene set may provide labeling for the evaluation of probiotics-based treatment.

## Highlights

• PR-enriched pit mucous cells drive the inflammation-to-cancer transition.
• Gene set was rigorously validated across multiple external cohorts.
• Probiotics modulate the gastric cancer-associated tumor-immune microenvironment.

## Graphical abstract

**Figure:**
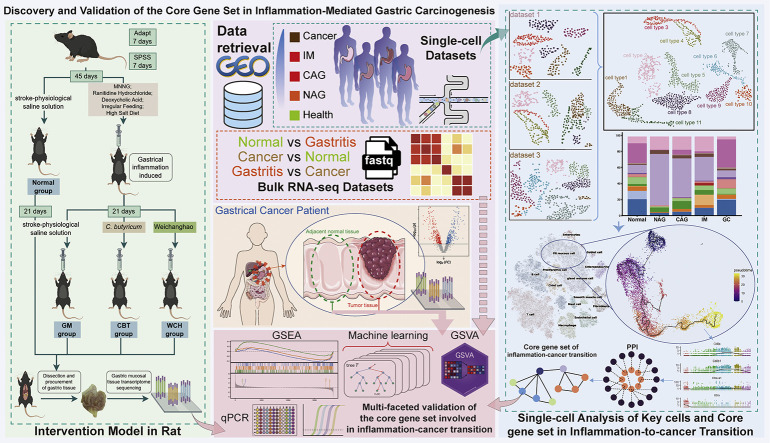

## 1 Introduction

Gastric cancer (GC) is a common and highly prevalent malignancies worldwide, with over one million new GC patients each year. It ranks fifth in incidence and fourth in mortality rate among all cancers worldwide [1]. The asymptomatic progression characteristics of GC lead to its initial diagnosis at an advanced stage, which diminishes the therapeutic efficacy of curative interventions and is correlated with adverse clinical outcomes. To reduce the incidence of GC and improve the prognosis of patients with gastric-cancer, preventive strategies are essential: primary prevention strategies include maintaining a healthy lifestyle and eradicating *Helicobacter pylori* infection, while secondary prevention focuses on enhancing the accuracy of clinical diagnosis, which is the most effective approach for improving patient outcomes [2]. Epidemiological data show that approximately 25 % of human cancers in the world are related to either chronic inflammation or chronic infection [3]. The strongest correlation between inflammation and cancer is demonstrated in the progression from gastritis, hepatitis, and cervicitis to GC, liver cancer, and cervical cancer, respectively. Treatments with anti-inflammatory drugs are available for improving the therapeutic effect [[4], [5], [6]]. Prolonged exposure to inflammatory factors (e.g., IL-6, IL-1α, INF-γ, TGF-β1) and reactive oxygen species (ROS) promotes epithelial DNA damage and mutagenesis [7]. The activation of the NF-κ and STAT3 pathways by inflammatory mediators in the microenvironment triggers the activation of genes including *MYC, BCL2, CCL2*, and *VEGF*, which cause cancer progression through cell proliferation and survival, angiogenesis, invasiveness, and metastasis [8]. Collectively, the insidious progression and inflammation-driven pathogenesis of GC highlight the critical potential of early interception strategies targeting premalignant inflammation as a superior therapeutic paradigm.

Cancer is now recognized as a multifaceted "ecosystem," where malignant tumor cells coexist and interact with a diverse array of non-cancerous cells. At the core of this ecosystem lies the tumor microenvironment (TME), a dynamic and heterogeneous assembly of cell types including immune cell subsets, cancer-associated fibroblasts (CAFs), endothelial cells, pericytes, and other tissue-specific residents, all of which play pivotal roles in tumor progression. The TME’s composition and functional state are far from uniform, and vary widely depending on the factors such as the organ of origin, the intrinsic properties of the cancer cells, the tumor’s stage, and individual patient characteristics [9]. Traditional bulk RNA-sequencing, which averages gene expression across the entire tissue sample, obscures the intricate interactions among these diverse cell populations within the TME. This limitation hampers a comprehensive understanding of the TME’s complexity, making it challenging to detect rare cell types or pinpoint specific genetic alterations at the cellular level. By contrast, single-cell transcriptomics represents a transformative advance, enabling unprecedented resolution for the interrogation of gene expression at single-cell resolution. This approach have been utilized to analyze the heterogeneity within tumors and has shed new light on the molecular mechanisms driving cancer development and progression. For example, Yang et al. [10] applied single-cell RNA sequencing to hepatocellular carcinoma (HCC), identifying 119 marker genes that distinguish malignant HCC cells from healthy liver tissue. Similarly, Li et al. utilized pseudotime trajectory analysis on single-cell RNA-sequencing data to trace colorectal cancer progression, uncovering 377 genes linked to the transition from normal to cancer cells [11]. Beyond primary tumors, this technology has shed light on metastatic processes, such as the signaling networks between the inflammatory CAFs and tumor-associated macrophages in bladder cancer that promote metastasis [12]. Comparative analyses of colorectal cancer and inflammatory bowel disease have revealed their common immune mechanisms, suggesting their involvement in the early events of the inflammation-to-cancer transition [13]. Using single-cell pseudotime trajectory analysis, Lu et al. found that CYP7A1+ hepatocytes drive NASH-associated liver cancer [14]. Collectively, single-cell RNA sequencing (scRNA-seq) is proving to be invaluable for mapping gene expression and cellular changes during cancer transformation, potentially identifying critical transcription factors as targets for early diagnosis and therapy [15]. Even though previous research has revealed the crucial roles of key factors in GC, its internal mechanisms remain unclear, mainly due to the high complexity and heterogeneity of cancerous tissue. Single-cell transcriptomics provide a more precise and in-depth analysis of the crucial factors associated with malignancy.

Dysbiosis of gastric microbiota plays a pivotal role in the progression from chronic gastritis to GC through multiple mechanisms [16]. Synergistic dysregulation of *Lactobacillus* and *Veillonella* is correlated with functional alterations that may promote carcinogenesis [17]. Dysbacteriosis characterized by the loss of beneficial taxa permits pathogenic overgrowth, which synergistically drives mucosal inflammation [18]. Clinical studies show that long-term use of non-steroidal anti-inflammatory drugs and anti-infective drugs directly affects the composition and function of gastrointestinal flora [19]. Emerging probiotic-based therapeutics provide a dual-functional strategy to restore gut microbial homeostasis through competitive exclusion of pathogens and reinforcement of intestinal barrier integrity, while simultaneously exerting anti-inflammatory effects. Probiotics or probiotic-derived bacteriocins can directly interact with COX2, modulating inflammatory NLRP3, NF-κB, PI3K/AKT and caspase pathways which activate autophagy and apoptosis. Previous probiotic interventions for disease have been largely empirical, obtaining clinically heterogeneous outcomes due to a lack of defined mechanistic targets [20]. It is necessary to carry out probiotics-based interventions that target the mechanisms of disease occurrence and development, thereby enabling a rigorous evaluation of their therapeutic efficacy.

In this study, we addressed two critical challenges in GC prevention: (1) the lack of reliable biomarkers for early detection of the gastritis-to-cancer transition, and (2) the absence of standardized criteria for the evaluation of probiotic interventions. To overcome these limitations, we integrated single-cell transcriptomics with multicohort validation to identify the core regulatory networks driving inflammation-associated malignancy. We then assessed the precancerous intervention potential of *C. butyricum* and Weichanghao® synbiotics by blocking the activation of tumorigenesis-related pathways.

## 2 Materials and methods

### 2.1 Data collection and quality control

Datasets GSE134520, GSE183904, and GSE163558 were downloaded from the Gene Expression Omnibus (GEO) database (https://www.ncbi.nlm.nih.gov/geo/), and samples were selected from these data sets to constitute the dataset for this study, which included 3 non-atrophic gastritis (NAG), 3 chronic atrophic gastritis (CAG), 6 intestinal metaplasia (IM), 9 Normal, and 10 GC samples as detailed in Supplementary Table 1. The raw single-cell counts table of all samples were read using the Python package Scanpy [21], followed by quality control including cell filtering-only cells which met all of the following threshold were selected to proceed to the next step): 1) mitochondrial gene expression ratio >20 %, 2) number of expressed genes between 500 and 3000, and 3) no predicted doublets by Scrublet [22]-and gene filtering-genes expressed in <3 cells were filtered (Supplementary Figs. 1 a, b). Following filtering, the count matrix was normalized using a shifted-log transformation to stabilize variance. Then, a regression-based correction was applied to remove the linear effects stemming from mitochondrial gene expression, and the total Unique Molecular Identifier (UMI) counts were subsequently scaled to a standard normal distribution. For principal component analysis (PCA), the top 4000 most highly variable genes were identified. Batch effects between the samples were removed using Harmony.

### 2.2 Cell clustering and annotation

The t-SNE and UMAP were used for further dimensionality reduction to enable visualization. Cells were clustered using the Leiden algorithm with resolution 1. The cell type annotation of these clusters was manually performed by examining the expression patterns of the relevant marker genes using the pathway_aucell_enrichment function in OmicVerse [23] (Supplementary Table 2 and Supplementary Figs. 1 c, d). Differential abundance analysis was performed using the pertpy.tl.Sccoda function from the pertpy [24] package applied to identify the differences in the cellular composition between various groups (Supplementary Fig. 1 e). The differential genes of each cell cluster were obtained using the rank_genes_groups function in Scanpy with the Wilcoxon test. The pathway_enrichment function in Omicverse was then used to perform Gene ontology (GO) enrichment analysis on the differential genes of the cell clusters to validate the rationality of the cell clustering.

### 2.3 Identification of transitional cells

CopyKAT [25] was employed to predict the copy number states of epithelial cells, thereby identifying tumor cells. The cells previously annotated as pit mucous cells (PMC), which were predominantly populated by malignant cells, were subseted and re-clustered into 8 subclusters. Then, the CytoTRACE2 [26] within the OmicVerse framework was used to predict the developmental trajectories of the PMC cells and to classify and score their developmental potential.

### 2.4 Identification of inflammation-cancer transitional genes

We applied the graph_test function to identify the candidate genes and calculate their Moran's I values on the Monocle3 trajectory. Then, gene set enrichment analysis (GSEA) [27] was performed by using the clusterProfiler [28] R package based on the rank of Moran's I values of the candidate genes to evaluate the key pathways involved in the inflammation-to-cancer transition (Supplementary Fig. 2 b). Genes exhibiting a Moran's I index greater than 0.01 with a statistically significant spatial autocorrelation (*p* < 0.001) were selected as input for the STRING database [29] to predict protein interactions among the candidate genes (minimum required score: 0.7). The top 100 genes with maximal node degrees in protein-protein interaction network were identified as the core genes in the inflammation-to-cancer transition (Supplementary Figs. 2 a, c).

### 2.5 Verifying inflammation-to-cancer transitional genes

The classification performance of this gene set in distinguishing between different conditions was evaluated using the random forest classification model from scikit-learn, across the datasets GSE5081 (normal mucosa vs. inflammatory mucosa), GSE79973 (normal mucosa vs. cancerous mucosa), and GSE55696 (inflammatory mucosa vs. cancerous mucosa). Utilizing the leave-one-out cross-validation strategy for cohort-wise dataset partitioning, the optimization of hyperparameters was conducted via 10-fold cross-validation during model training. This approach ensured a robust assessment of the model's performance across the partitioned cohorts. This strategy ensured a rigorous and reliable assessment of the classifier’s performance across diverse cohorts. The generalizability of the diagnostic model across validation cohorts was confirmed by the receiver operating characteristic (ROC) curve, achieving area-under-the-curve (AUC) values between 0.7 and 0.9. Additionally, we collected clinical samples (Ethical approval No: 20,220,207) of GC and adjacent tissues from patients with cancer and performed transcriptome sequencing to validate the expression patterns of the key core inflammation-to-cancer transition gene set. Human clinical sample collection was conducted in accordance with the Declaration of Helsinki.

### 2.6 Animals and experimental design

All animal experiments strictly complied with ARRIVE guidelines and the U.K. Animals (Scientific Procedures) Act 1986 (Approval No: SYDWLL-2022–028​​).Specific-pathogen-free (SPF) Wistar male rats (4 weeks old) were purchased from Jinan Pengyue Experimental Animal Breeding Co. Ltd. and housed at Qingdao East Sea Pharmaceutical Co., Ltd. *C. butyricum* CGMCC0313.1 broth and Weichanghao (WCH, a commercial probiotic product containing *Weizmannia coagulans* TBC169, prebiotics​, and Chinese herbal medicine) were provided by Qingdao East Sea Pharmaceutical Co., Ltd. The rats were kept at 20∼25 °C and 40–70 % humidity with 12 h of alternating light and dark. SPF-rat maintenance diet was provided with sterile bedding changed at least twice a week. After adaption for 7 d, the rats were divided into four groups: normal control group (NC), gastritis model group (GM), *C. butyricum* treatment group (CBT, 3.94 × 10^9^ CFU/kg BW), and Weichanghao treatment group (WCH, 3.13 g/kgBW). Gastritis was induced by the following combination of methods: N-methyl-N'-nitro-N-nitrosoguanidine (MNNG), ranitidine hydrochloride, sodium deoxycholate, hunger and satiety disorder, and high-salt diet. Every 3 days constituted a modeling cycle. For the first 2 days, the rats were orally infused with MNNG (30 mg/kg BW) and ranitidine hydrochloride (20 mg/rat) accompanied by free feeding. On the third day, the rats were orally infused with MNNG (30 mg/kg BW) and sodium deoxycholate (8 mg/rat) accompanied by fasting. Sterilized water containing 1.5 % sodium chloride was used as drinking water. The NC group was given sterile normal saline by gavage along with routine feeding.

### 2.7 RT-qPCR analysis

Morphological changes of the gastric mucosa were observed and photographed. Total RNA was extracted from gastric tissue using the RNA-easy Isolation Reagent Kit (Vazyme, Nanjing, China). RNA quality was assessed via 1 % agarose gel electrophoresis, and concentrations were measured using a spectrophotometer (TECAN, Austria). Total RNA was reverse-transcribed using HiScript IV RT SuperMix for qPCR (Vazyme, Nanjing, China). The primer sequences for the inflammatory factors were as follows: *GADPH* (F: GGCACAGTCAAGGCTGAGAATG, R: ATGGTGGTGAAGACGCCAGTA), *IL-1α* (F: GTAAGAGAAGAGCAAAGCC, R: CAACTTTATCCTACCCATCCG), *IL-6* (F: AGAGACTTCCAGCCAGTTGC, R: AGTCTCCTCTCCGGACTTGT), *IFN-γ* (F: ACTGCCAAGGCACACTCATT, R: AGGTGCGATTGGATGACACT), *TGF-β1* (F: AGGCGGTGCTCGCTTTGTA, R; GTTGCGGTCCACCATTAGC). The operating program for the 20 μL reaction system was set as 95 °C, 60 s; 95 °C, 60 s; and 60 °C, 20 s (40 cycles).

### 2.8 RNA-seq and enrichment analysis

The human key core inflammation-to-cancer transition genes were mapped to rat orthologs using cross-species homolog gene mapping via HGD [30] (Supplementary Table 5). Subsequently, and gene set variation analysis (GSVA) [31] was applied to assess the expression levels of this gene set across various intervention groups in rats. Quality control was performed using fastp [32] to filter low-quality reads, remove adapters, and trim reads. In addition, reads were aligned to the rat reference genome (Rattus_norvegicus.mRatBN7.2 and Homo_sapiens.GRCh38 from ensembl) using the STAR [33] aligner. FeatureCounts [34] from Subread was then used to generate a gene-level counts matrix based on the annotation files (Rattus_norvegicus.mRatBN7.2.112 and Homo_sapiens.GRCh38.112 gtf files). Differential expression analysis was conducted using DESeq2 [35], GSVA and GSEA were performed with the ClusterProfiler package to identify significant pathways and gene sets.

## 3 Results

### 3.1 Single-cell transcriptional map of disease progression in GC

**Fig. 1:**
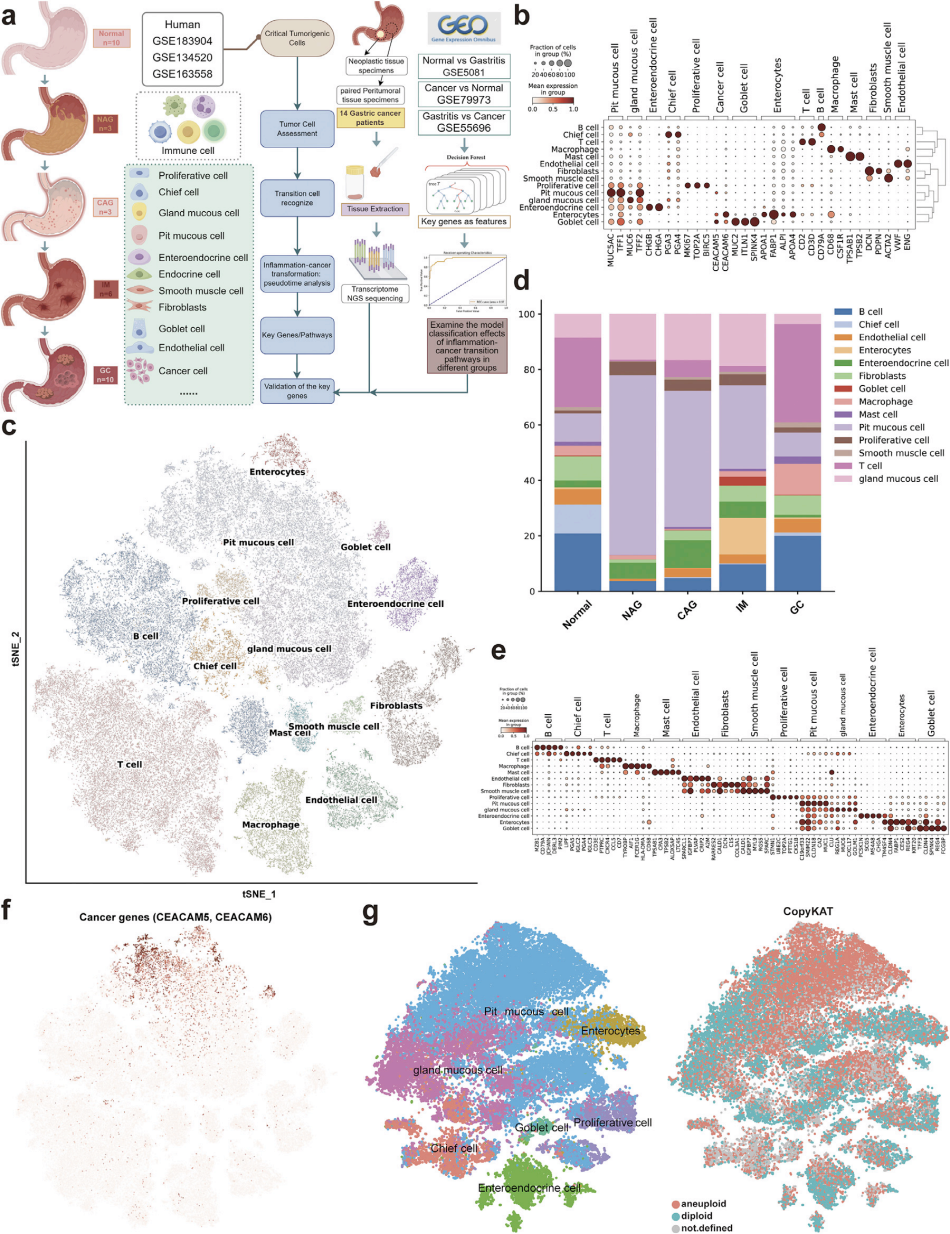
Analysis of stomach samples using scRNA-seq at different stages of gastric cancer. **a.** Diagrammatic depiction of data collection. **b.** The bubble plot shows the expression of marker genes in different cell types, where the color depth indicates the average expression, and the point size indicates the proportion of marker genes expression. **c.** UMAP plot showing the cell types. **d.** Cell type composition in different stages. **e.** Expression profiles of five characteristic genes in different cell types. **f.** Distribution of oncogenes in different cell populations. **g.** Re-clustering of epithelial cells and calculation of gene copy number.

To investigate the key cells and genes in gastric inflammation-to-cancer progression, we analyzed three scRNA-seq datasets (GSE183904, GSE134520, GSE163558). These included normal tissue (*n* = 11), NAG (*n* = 3), CAG (*n* = 3), IM (*n* = 7), and GC (*n* = 9), representing the full disease spectrum (Fig. 1a). After removing low-quality cells and low-expression genes, a total of 113,049 cells and 30,004 genes that passed quality control were retained for subsequent analysis. Following standardized preprocessing steps including normalization, dimensionality reduction, and batch effect correction using Harmony, the cells were subjected to unsupervised clustering and categorized into 14 cell types based on marker gene expression. These cell types included PMCs (*MUC5AC, TFF1*), gland mucous cells (*MUC6, TFF2*), enteroendocrine cells (*CHGB, CHGA*), chief cells (*PGA3, PGA4*), proliferative cells (*MKI67, TOP2A, BIRC5*), goblet cells (*MUC2, ITLN1, SPINK4*), enterocytes (*APOA1, FABP1, ALPI, APOA4*), T cells (*CD2, CD3D*), B cells (*CD79A*), macrophages (*CD68, CSF1R*), mast cells (*TPSAB1, TPSB2*), fibroblasts (*DCN, PDPN*), smooth muscle cells (*ACTA2*), and endothelial cells (*VWF, ENG*) (Fig. 1b, c). The top three cells by number in the cell atlas were the T cell, PMC, and B cell. We observed significant differences in the composition of the cell types at different stages (Fig. 1d). For instance, IM exhibited enterocyte enrichment, including enterocytes and goblet cells and concurrently developed a gradual reduction in PMC population throughout disease progression from NAG to GC. Interestingly, an explosive increase in PMC was observed during the transition from normal to NAG, which was consistent with the results obtained in previous studies [36], suggesting that the development of the inflammation-cancer transition was significantly influenced by dynamic changes in the cell composition. We calculated top five differentially expressed genes for each subpopulation, which verified the robustness and precision of cell type annotation (Fig. 1e). The genes *C19orf33, SMIM22, CLDN18, CA2*, and *MUC1* exhibited specific overexpression in PMCs. Notably, *C19orf33* was closely associated with tumor cell immortalization [37], while *CLDN18* which was highly expressed in GC has been identified as a potential therapeutic target [38]. Furthermore, MUC1 has been shown to promote proliferation and invasion in multiple cancer types [39]. GC samples showed high expression of the *CEACAM5* and *CEACAM6* cancer genes. Subsequent analysis of the oncogenic genes enrichment across subpopulation datasets revealed that epithelial cells exhibited the highest enrichment of cancer-associated genes, as shown in Fig. 1f. Previous studies had also demonstrated that the malignant transition (e.g. epithelial-mesenchymal transition) of epithelial cells promotes cancer progression [[40], [41], [42]]. Epithelial cells were re-clustered and annotated into 8 cell subpopulations. Single-cell copy number variation analysis using CopyKAT revealed that tumor cells were specifically enriched in PMCs clusters. These findings established PMCs as the principal cellular component of the gastric epithelium, simultaneously indicating their potential role as cellular origins of carcinogenesis (Fig. 1g). Consequently, PMCs were selected as the focal cell population for the subsequent investigations of tumorigenic development and disease progression.

### 3.2 Identification of transitional cells related to inflammation-to-cancer transition

**Fig. 2:**
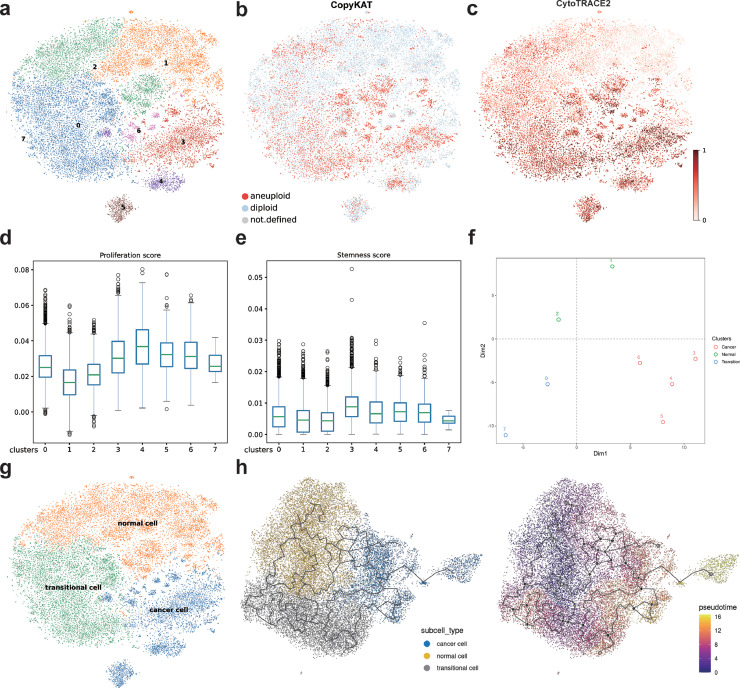
Re-clustering analysis of pit mucosal cells (PMCs). **a.** Re-clustering of PMC into eight subclusters. **b.** UMAP visualization showing identification of malignant cells using CopyKAT. Red dots represent aneuploid malignant cells, blue dots represent diploid normal cells, and gray dots represent unclassified cells. **c.** Pseudotemporal ordering based on Cytotrace2 reveals cellular developmental differentiation trajectories, with color saturation corresponding to developmental plasticity. **d.** Boxplot displaying the proliferation score for each cluster. **e.** Boxplot displaying the stemness score for each cluster. **f.** PCA visualization of expression similarity in various cellular subgroups through dimensionality reduction. **g.** UMAP plot showing the distribution of annotated normal, transitional, and cancer cells. **h.** Pseudotime reconstruction of inflammation-cancer transition in gastric PMC using Monocle3.

To elucidate the genes and biological processes driving the gastric inflammation-to-carcinoma progression, we subsequently focused on PMCs for in-depth analysis. Based on independently re-clustering the pre-annotated PMC populations into eight distinct subclusters (Fig. 2a), we facilitated a framework for systematic spatial investigation to resolve the transcriptional dynamics across the pathological continuum from non-atrophic gastritis to gastric carcinoma. CytoTRACE2 and UMAP were used to infer differentiation potential and visualize developmental trajectories (Fig. 2c). Trajectory analysis showed developmental initiation in clusters C1 and C2, and predicted malignant cells to be predominantly localized at clusters C3, C4, C5, C6, C0, and C7 (Fig. 2b), demonstrating progressive spatial consolidation along the differentiation pseudotime. Complementary functional profiling based on proliferation and stemness scoring (Supplementary Table 2) identified diminished scores in progenitor clusters C1-C2 in contrast with elevated scores in advanced clusters C3-C6 (Fig. 2d, e, Supplementary Fig. 3 b). Combined with the spatial distribution of cellular subpopulations in the expression-based PCA dimensionality reduction (Fig. 2f), C1 and C2 were annotated as normal cells, C0 and C7 as transitional cells, and the remaining clusters (C3-C6) as cancer cells (Fig. 2g, Supplementary Fig. 3). Feature genes (Supplementary Fig. 2 a) indicated that normal cells exhibited high expression of *GKN2* and *GKN1* which are involved in the regulation of gastric mucosal homeostasis [37]. Cancer cells exhibited elevated levels of *HSP90AA1* and *ZFP36L2*, which facilitated tumor progression and oncogenesis, thereby enhancing cancer cell aggressiveness [38,39,43,44]. Transitional cells were in the intermediate process of carcinogenesis, and the expansion of this population may increase the likelihood of gastric PMC changing from normal to cancer. Leveraging prior pseudotemporal developmental trajectory and malignancy prediction analyses, pseudotime analysis was conducted on PMC using Monocle3, with normal cells set as the trajectory starting point (Fig. 2h). Genes displaying both temporal correlation and positive spatial autocorrelation (Moran's I; Supplementary Table 3) were then computationally identified. GSEA based on Moran's I revealed significant enrichment of key signaling pathways along the trajectory (Supplementary Fig. 2b), especially for the PI3K-Akt, cytokine-cytokine receptor interaction, and chemokine signaling pathways, suggesting their potential role in driving cellular carcinogenesis.

For significant spatial autocorrelated genes, a protein-protein interaction network was constructed using the STRING database (topological visualization presented in Supplementary Fig. 2a). A core gene set associated with oncogenic transformation was identified (Supplementary Fig. 2c, Supplementary Table 4). Notably, this gene set demonstrated predominant enrichment in ribosomal protein family members. Finally, the core gene set involved in the transformation from gastric inflammation to cancer was selected from these candidate genes. Furthermore, the core genes in the set were further investigated and validated.

### 3.3 Identification and verification of genes related to inflammation-to-cancer transition

**Fig. 3:**
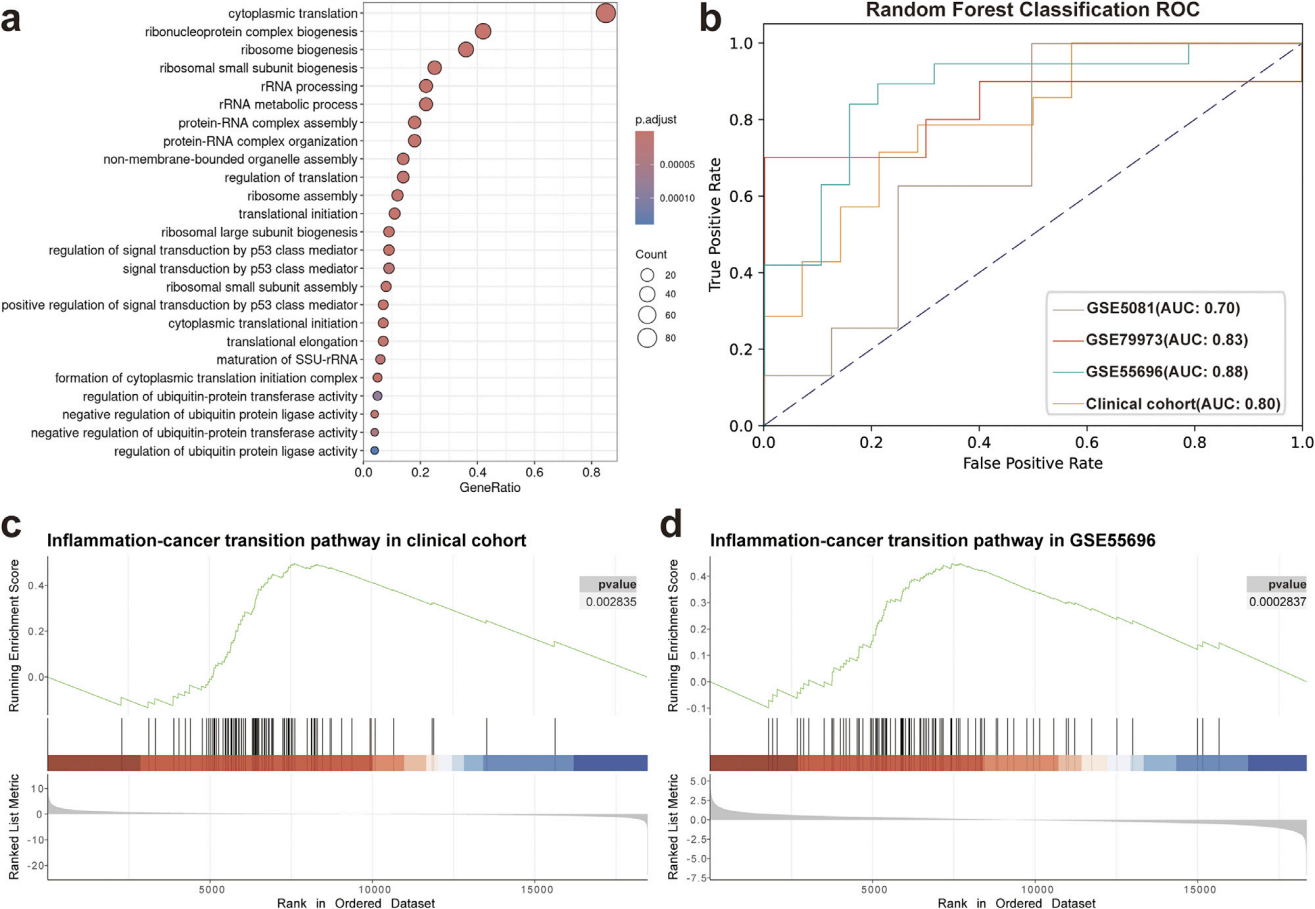
Enrichment pathways of core gene sets and validation from clinical sample. **a.** Core gene set KEGG enrichment pathway. **b.** ROC curve derived from a multicohort random forest model constructed using core gene signatures of the inflammation-to-cancer transition. The model development incorporated leave-one-out (LOO) cohort partitioning for training-validation splits with subsequent 10-fold cross-validation optimization. **c.** GSEA enrichment plot of the predicted core genes in clinical samples. **d.** GSEA enrichment plot of the predicted core genes in external datasets.

Using over-representation analysis of the core gene set associated with inflammation-to-cancer transition, 25 significantly enriched pathways were identified (adjusted p-value < 0.05) (Fig. 3a). The top-ranked pathways included cytoplasmic translation (GO:0002,181), ribonucleoprotein complex biogenesis (GO:0022,613), and ribosome biogenesis (GO:0042,254), suggesting that these biological processes may drive cellular reprogramming during cancer initiation and progression (Fig. 3a). More importantly, the coordinated enrichment of translational and ribosome assembly pathways implied potential dysregulation of protein synthesis homeostasis in the inflammatory-cancer transition. To further validate the clinical relevance of the core gene signature, cross-platform validation analysis was conducted using four independent GC transcriptomic datasets. These datasets were composed of three publicly available cohorts (GSE5081, GSE79973, and GSE55696) and an in-house cohort.

The in-house cohort was comprised of paired tumor and adjacent normal gastric tissue samples from 14 patients with GC for meta transcriptomic sequencing analysis. Using paired differential expression analysis, 2177 significantly dysregulated genes (FDR<0.05) were identified, comprising 1175 upregulated and 1002 downregulated candidates in tumor tissues (Supplementary Fig. 4a). Gene set enrichment analysis revealed significant enrichment of oncogenic pathways (e.g., PI3K-Akt signaling) and inflammatory response pathways (e.g., IL-17 and TNF signaling) in the tumor microenvironment (Supplementary Fig. 4b). Notably, these activated pathways showed strong correlations with tumor progression markers and immune cell infiltration patterns.

Furthermore, to validate the expression changes of the core gene set across different stages of GC progression, we collected external prospective clinical cohorts including specimens of normal tissue, gastritis, and gastric adenocarcinoma. The random forest classifier model was constructed using core genes as discriminative features in multi-cohort. The random forest classifier demonstrated robust performance across cohorts (Fig. 3b), thereby demonstrating the effectiveness of the identified core genes in distinguishing gastric tissue samples at different disease stages. Notably, GSEA identified significant upregulation patterns (*p* < 0.001, FDR < 0.05) of the 100-core-gene panel across critical pathological transitions: normal (peritumoral GC) vs. GC, and gastritis vs. GC (Fig. 3c, d). These results confirmed the biological validity of the inflammation-cancer transition signature as a potential biomarker for treatment response evaluation.

### 3.4 Effect of *C. butyricum* and synbiotics on gastric inflammation-to-cancer transition

**Fig. 4:**
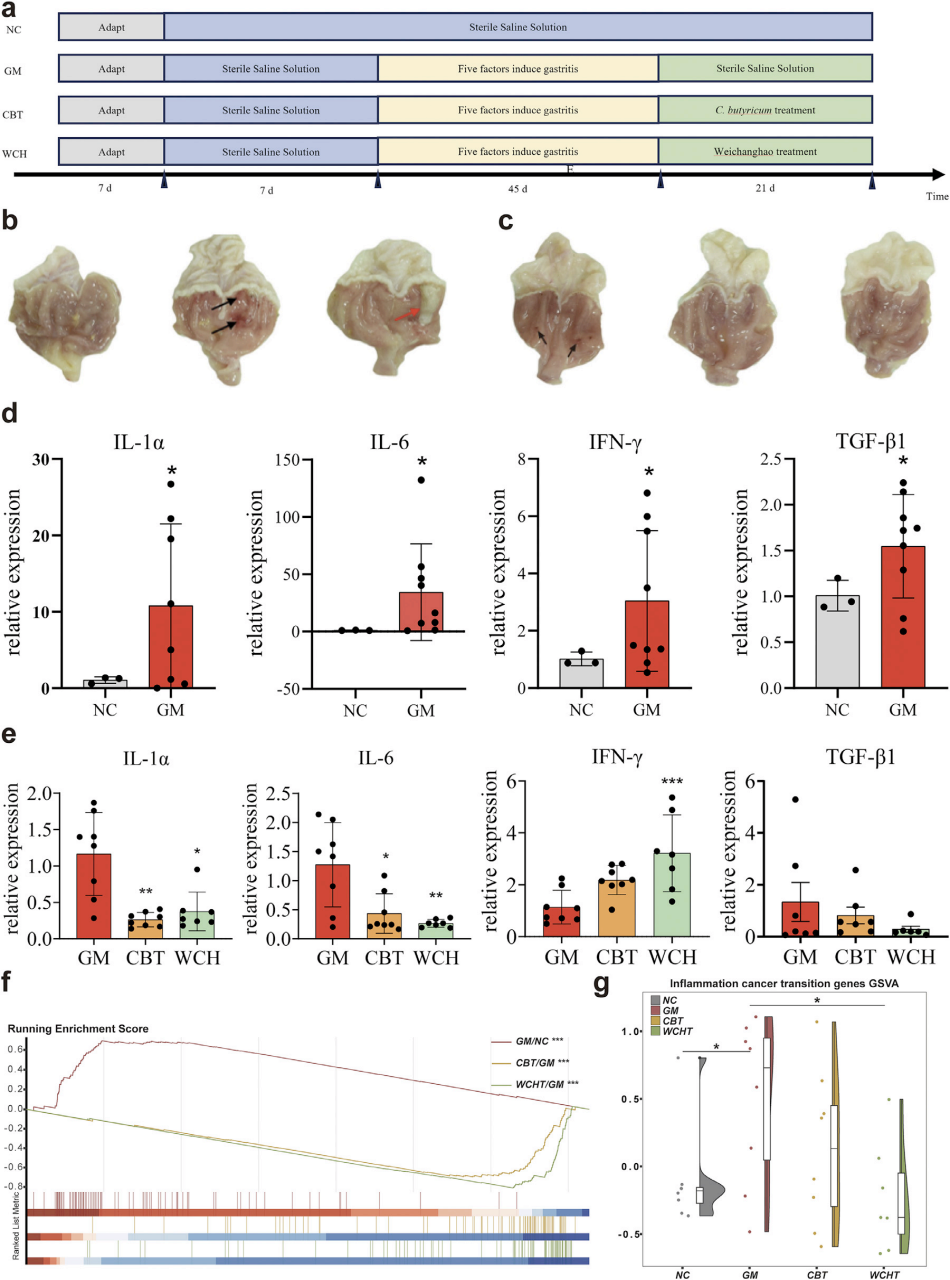
Influence of intervention on gastritis and the inflammation-to-cancer transition. **a.** Outline of experimental plan. **b.** Gastric mucosa status of rats during gastritis model construction. The black arrow indicates gastric hemorrhagic macules, and the red arrow indicates gastric ulcer. **c.** Gastric mucosa of rats after intervention. **d.** Expression of inflammatory factors in gastric tissues of rats with gastritis. **e.** Expression of gastric inflammatory factors after intervention. **f.** GSEA analysis of the predicted core genes set in different rat groups. **g.** GSVA analysis of the predicted core genes set in different rat groups. Statistical significance was determined by *t*-test. *p < 0.05; **p < 0.01; ***p < 0.001. GM: gastritis model. CBT: *C. butyricum* treatment. WCH: synbiotics (Weichanghao) treatment.

Based on the identified inflammation-to-cancer transition marker genes, we established a gastritis rat model followed by intervention protocols to assess their therapeutic potential on gastric neoplasia. The gastritis rats were treated with *C. butyricum* and synbiotics (WCH), respectively, and the modeling process is shown in Fig. 4a. The gastric mucosa in the gastritis model (GM) group was observed as shown in Fig. 4b The gastric mucosa in the control group exhibited an intact, glossy epithelial surface without histopathological hemorrhagic macules, while by contrast, the GM group presented marked mucosal irregularities, including petechial hemorrhages and even focal ulcerative erosions (Fig. 4b). The relative expression levels of four inflammatory factors (*IL-1α, IL-6, INF-γ, TGF-β1*) in the gastric tissue of gastritis rats were significantly increased (Fig. 4d).

After 3 weeks intervention, the gastric mucosa status of rats was examined (Fig. 4c). In both groups treated with either *C. butyricum* and synbiotics Weichanghao (CBT and WCH), the mucosa showed significant improvement, with no hemorrhagic lesions and a smooth surface maintaining physiological elasticity. Expression of *IL-1α, IL-6* and *TGF-β1* decreased after interventions (Fig. 4e). *C. butyricum* and WCH synbiotics inhibited gastritis by improving the mucosal status and reducing the release of inflammatory factors. However, probiotic intervention unexpectedly elicited a significant upregulation of *IFN-γ*, in contrast with the overall anti-inflammatory trend observed in other inflammatory factors (*IL-1α, IL-6, IL-10,* and *TGF-β1*). This upregulation of *IFN-γ* may reflect a biphasic modulation of immune responses [45]; however, the precise mechanism remains elusive. Furthermore, transcriptome sequencing was performed on the gastric tissues of rats after intervention to analyze the expression of genes related to the inflammation-to-cancer transition (Fig. 4f, g). Based on the core inflammation-to-cancer transition genes established, we systematically evaluated the modulatory effects of *C. butyricum* and WCH interventions on these molecular markers in rats. Comparative GSEA across different treatment groups showed that GM rats exhibited enrichment of core gene sets compared to normal rats, indicating that gastritis may progress to malignancy. After intervention with either *C. butyricum* or WCH, the genes related to the inflammation-to-cancer transition were significantly downregulated, demonstrating the therapeutic potential of both treatments in halting gastric malignant transformation. GSVA analysis corroborated this effect (Fig. 4g). In summary, the core genes demonstrated potentially predictive reliability for inflammation-to-cancer transition, as evidenced by the concordant validation in both clinical cohorts and preclinical animal models.

## 4 Discussion

The close relationship between chronic inflammation and cancer has been widely recognized. Given the multifactorial etiology of GC, development of target appropriate prevention strategies remains challenging. Consequently, early detection and clinical management of precancerous gastric lesions have emerged as a critical approach for reducing disease incidence and progression. This study innovatively delineated multi-stage cellular and molecular alterations along the gastric inflammation-to-cancer transition axis through scRNA-seq profiling of clinical specimens (normal, NAG, CAG, IM, GC) from GSE datasets, coupled with functional validation in probiotics-based treatment rat models. ScRNA-seq approach identified evolutionary gene networks driving inflammation-cancer transition, while interventions revealed that microbiome modulation effectively disrupted critical inflammatory-cancer crosstalk, demonstrating potential for intercepting premalignant progression.

We identified malignant transformation of PMCs, a subclass of epithelial cells, as an important factor in the development of GC. Kim et al. [46] pointed out that the reduced proportion of PMCs suggested either functional loss or their transformation into more aggressive cell types (e.g., IM-associated cells) during GC progression. IM is the intermediate stage of the transition from chronic gastritis to GC, which is manifested by the replacement of gastric mucosal epithelial cells by intestinal epithelial-like cells. Macrophages and fibroblasts were considered to promote inflammation and epithelial-mesenchymal transition through the B2M-TFRC/HLA-F axis and activation of SDC1 during the dynamic changes of the inflammatory microenvironment [47,48]. Zhou et al. [41] revealed the synergistic interplay between the differentiation status of cancer cells and immune microenvironments in GC. *IFN-α* and *INF-γ* response genes were expressed more specifically in immune-rich malignant cells than in immune-poor malignant cells. Subsequently, we identified transitional cells from PMC, according to stemness and proliferation, using CopyKat; these cells were characterized by high expression of Ribosomal protein such as *RPL34, RPS18*, and *RPS19*. The co-activation of cytoplasmic translation and ribosome synthesis pathway in transitional cells suggests a state associated with rapid cellular growth, differentiation, or repair, and pathologically, indicating uncontrolled proliferation and a preneoplastic state [49]. Furthermore, we identified 100 core genes in transitional cells based on their developmental trajectories and interaction networks. This gene set exhibits a significant enrichment of ribosomal proteins family (e.g., *RPS3A, RPS6, RPS8, RPS11, RPS19*) and translation-regulatory genes (e.g., *EEF1A1, EEF2, EIF3H, EIF4A1*), which sustained tumor cell growth and proliferation [50]. Ban et al. [51] also revealed that the epithelial-to-mesenchymal transition of tumor cells depends on increased ribosome biogenesis and the ensuing nascent protein synthesis driven by ERK and mTOR. Validation across external datasets and rat model of gastritis demonstrated that the core gene set achieved high accuracy (AUC > 0.7) in distinguishing gastric disease stage stratification, with upregulation in GC samples. Thus, we have established a model for early cancer diagnosis and assessment criteria of premalignant therapeutic intervention.

Our intervention study demonstrated that both *C. butyricum* and Weichanghao significantly suppressed pro-inflammatory factors and core genes associated with inflammation-to-cancer transition. IL-1α, IL-6, and TGF-β1 were important inflammation-promoting molecules, and previous studies have confirmed that they play a promoting role in the proliferation, migration, mesenchymal transformation, and peritoneal metastasis of GC cells [52,53]. ​*C. butyricum* and Weichanghao exhibited distinct therapeutic profiles. Specifically, Weichanghao demonstrated superior efficacy in modulating inflammatory factors and suppressing inflammation-to-cancer transition genes, thereby effectively halting disease progression through multi-target regulatory mechanisms. Although probiotics were explored to enhance pathogen infection recovery via nutrient competition and short-chain fatty acid production [54], their therapeutic efficacy in advanced malignancies remains suboptimal compared to early-stage interventions [55]. Our study not only delineated the core gene regulatory networks driving gastritis-carcinoma transition, but also experimentally validated that the intervention strategies (*C. butyricum* and Weichanghao) can attenuate premalignant progression in rat models. We highlighted a paradigm shift, in which the blockade of targeted core genes during the premalignant phase may prevent carcinogenesis more effectively than late-stage treatment. Taken together, these findings collectively highlighted the potential of core genes as both diagnostic biomarkers for early carcinogenic detection and targets of therapeutic agents for preventing precancerous lesions.

## 5 Conclusion

In this study, we dissected the molecular landscape of the gastric inflammation-to-cancer transition using single-cell RNA sequencing. Subsequently, external cohorts and rat model were investigated to validate the inhibitory effects of *C. butyricum* and synbiotics on the inflammation-cancer transition process. Our analysis uncovered significant heterogeneity among immune and epithelial cell populations, especially PMCs contributing to the early carcinogenic events arising from chronic inflammation. Notably, we identified 100 core genes set as a critical mediator of this transition, providing a potential target for early diagnosis. Through targeted modulation using *C. butyricum* and synbiotics, we demonstrated a marked suppression of pro-inflammatory and pro-carcinogenic pathways, highlighting the potential of microbiome-based strategies for GC prevention. These findings not only advance the understanding of the mechanisms involved in inflammation-driven carcinogenesis but also provide actionable insights for early diagnosis and prevention of gastric precancerous lesions. Future studies should focus on validating these results in human cohorts and exploring the translational applications of these interventions in clinical settings.

## Data Availability Statement

Publicly available data was downloaded from the GEO database (https://www.ncbi.nlm.nih.gov/geo/). Experimental data in this study are available on request to the corresponding authors

## CRediT authorship contribution statement

**Minmin Hu:** Writing – original draft, Investigation, Formal analysis. **Shiyang Xu:** Writing – original draft, Investigation, Formal analysis. **Ruofei Xu:** Validation, Investigation. **Xiangjie Qi:** Resources. **Xiaofeng Yu:** Resources. **Jinqi Wang:** Investigation, Conceptualization. **Yige Li:** Investigation. **Yangyang Liu:** Validation. **Guiran Xi:** Validation. **Junbao Yu:** Validation. **Mei Shi:** Writing – review & editing, Supervision, Investigation.

## Declaration of Competing Interest

The authors declare that they have no known competing financial interests or personal relationships that could have appeared to influence the work reported in this paper.

## Appendix Supplementary materials

Image, application 1

Image, application 2
