# Supplementary material for: Analysis of single-cell RNA sequencing data to examine the gastric inflammation-to-cancer transition and evaluation of the effect of probiotic on precancerous lesions
Source: Eng Microbiol. 2025 May 9;5(3):100208. doi: 10.1016/j.engmic.2025.100208 (PMC12967842; doi:10.1016/j.engmic.2025.100208)
Supplement: Supplementary file 1 [file mmc1.pdf]

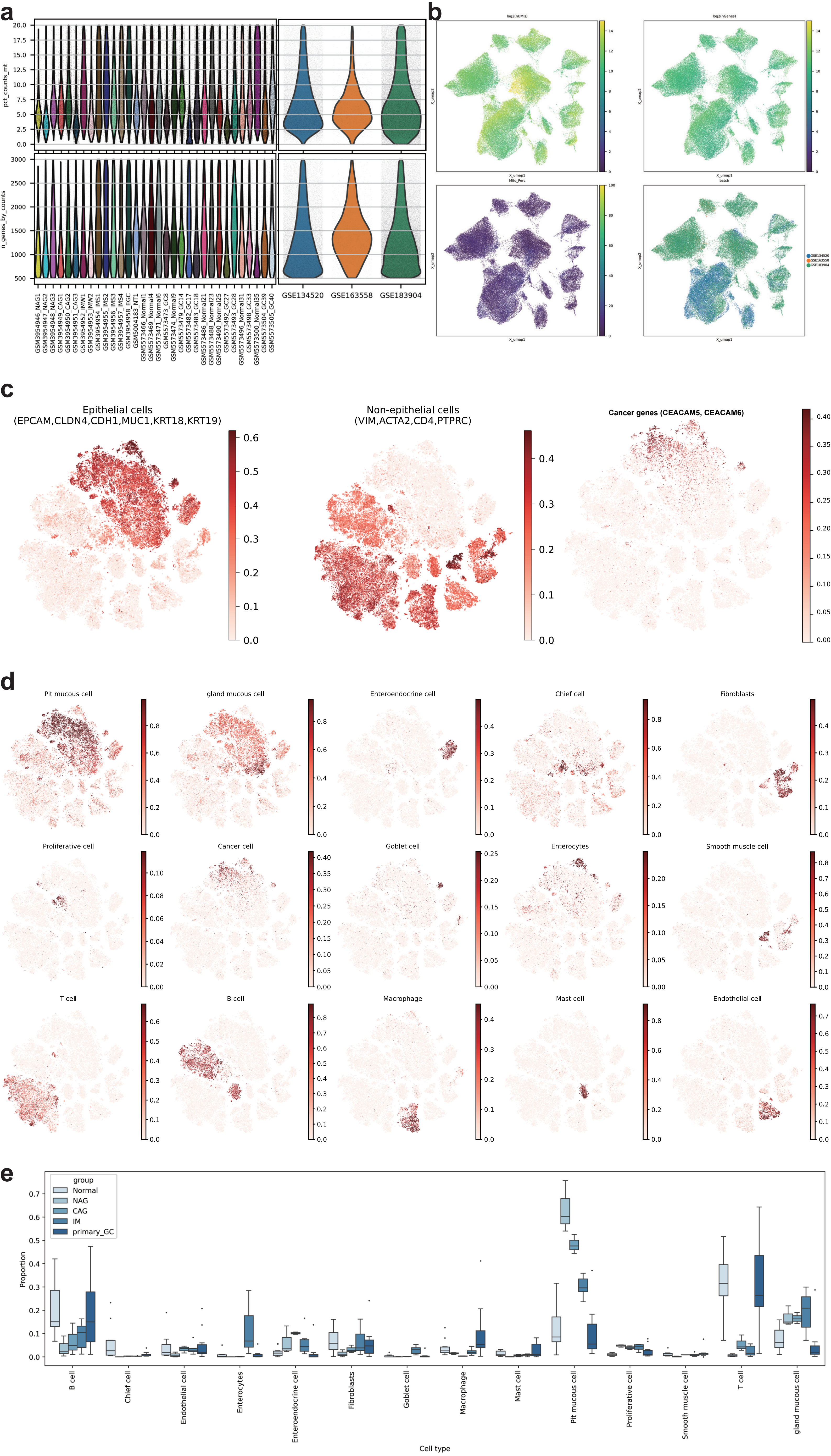

Supplementary Fig. 1 Single-cell omics data processing and cellular annotation. a, Violin plots illustrating post-quality-filtering metrics. Upper panel: Distribution of mitochondrial gene content (Mito_Perc) per cell. Lower panel: Total mRNA counts (nUMIs) and gene detection counts (nGenes). Left axis denotes biological samples; right axis represents integrated datasets. b, UMAP displaying feature distributions across datasets post-integration. Key metrics: nUMIs (total mRNA molecules per cell), nGenes (unique genes detected per cell), Mito_Perc (proportion of mitochondrial genes), and batch effects (different datasets). c, UMAP visualization of cell types defined by lineage-specific markers: Epithelial cells (EPCAM, CLDN4, CDH1, MUC1, KRT18, KRT19), non-epithelial cells (VIM, ACTA2, CD4, PTPRC), and carcinoma cells (CEACAM5, CEACAM6). d, UMAP dimensionality reduction map highlighting distinct cell type clusters. e, Comparative boxplots quantifying cell type proportions across experimental groups. Whiskers represent 1.5× interquartile range; center lines denote smedians.medians.

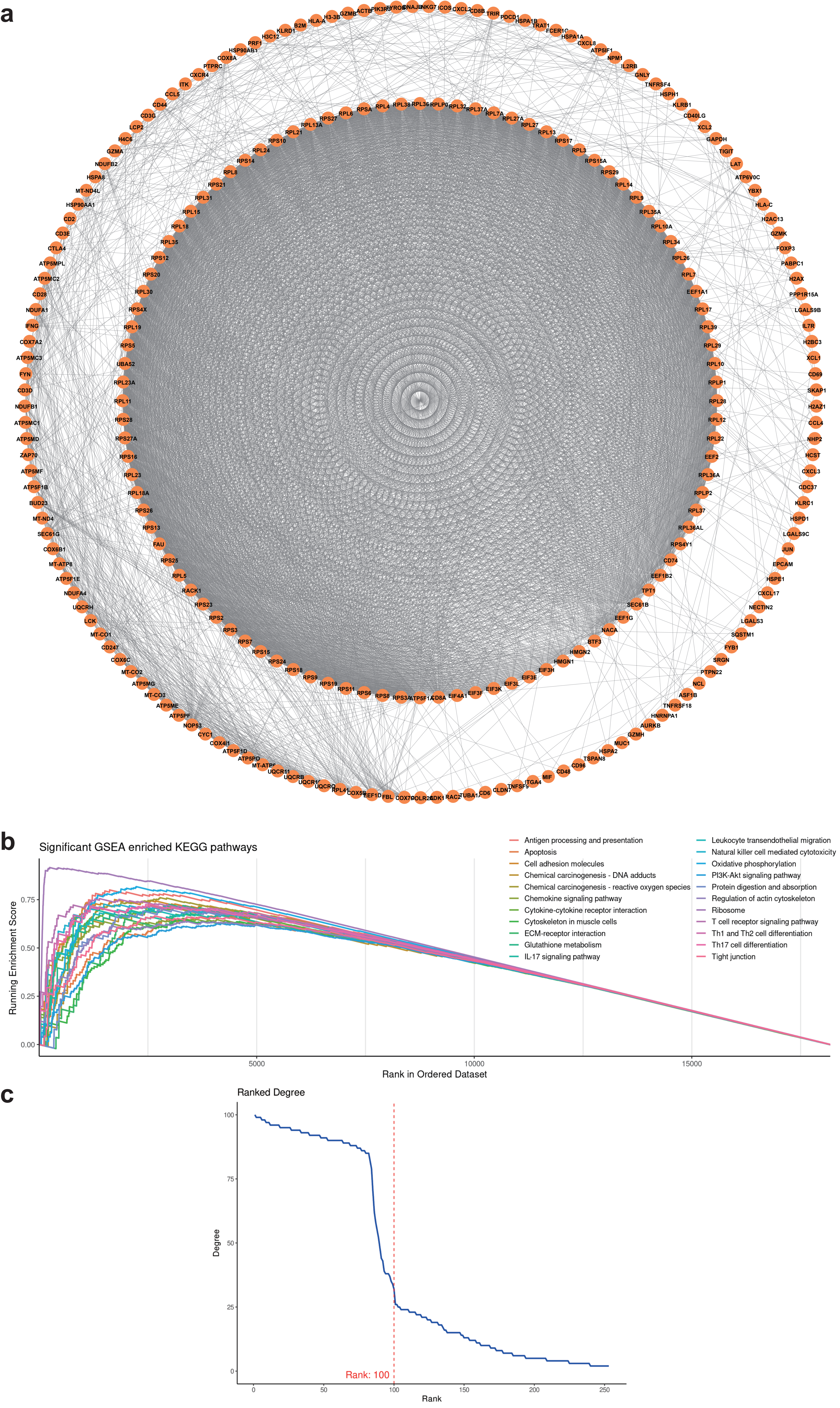

Supplementary Fig. 2 Core gene set screening and enrichment analysis. a, Protein-protein interaction (PPI) network of candidate genes screened through pseudotime analysis, constructed using the STRING database. b, Gene Set Enrichment Analysis (GSEA) of KEGG pathways ranked by Moran's I index, illustrating significantly enriched pathways (adjusted p-value < 0.001) associated with immune response, inflammatory regulation, and cancer-related mechanisms. c, Degree frequency distribution curve of nodes in the candidate gene PPI network, ranked by connectivity degree. The slope transition (red curve) at rank 100 indicates genes preceding this threshold form the core gene set.

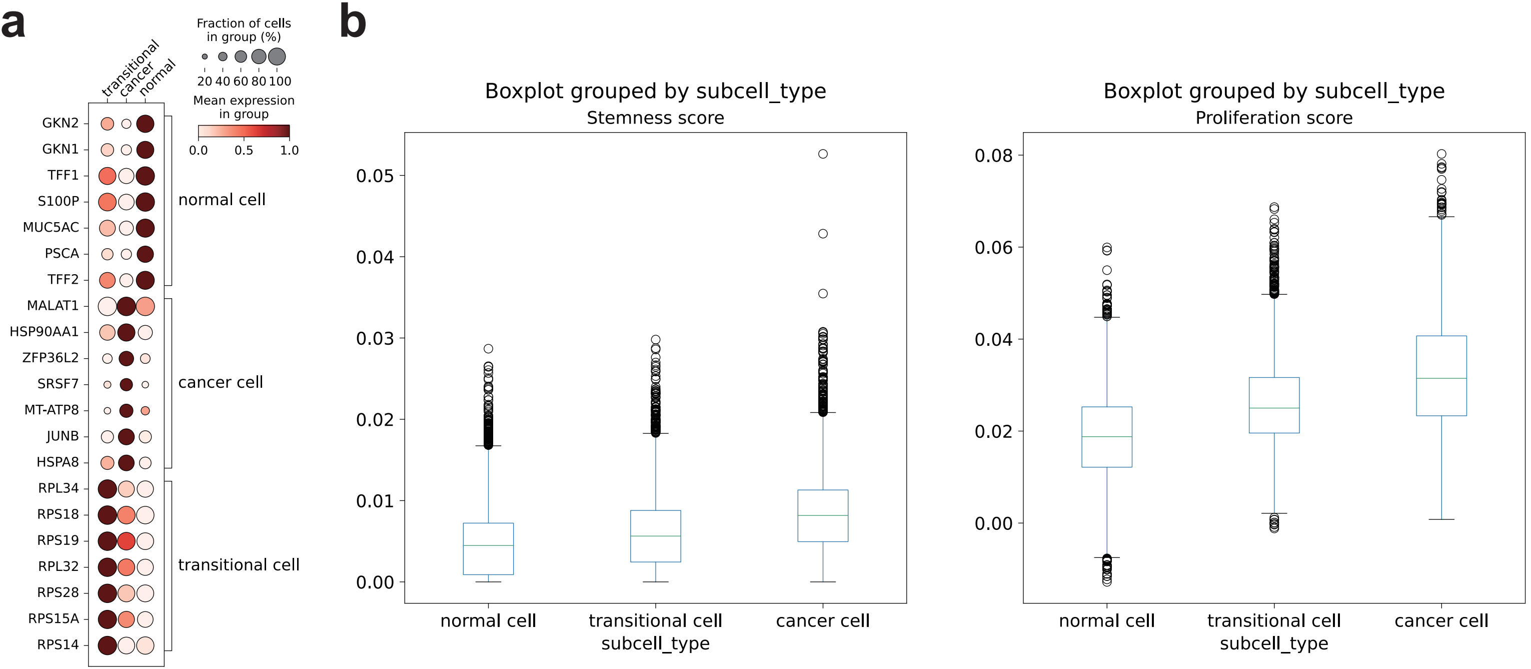

Supplementary Fig. 3 Expression profiles of cellular subpopulations during inflammatory-cancer transition in PMCs. a, Cell type-specific differentially expressed genes among normal, cancerous, and transitional cells in PMCs. b, Boxplot illustrating the stemness and proliferative gene expression scores across normal cells, cancer cells, and transitional-state cells in PMCs. Whiskers represent 1.5× interquartile range; center lines denote smedians.medians.

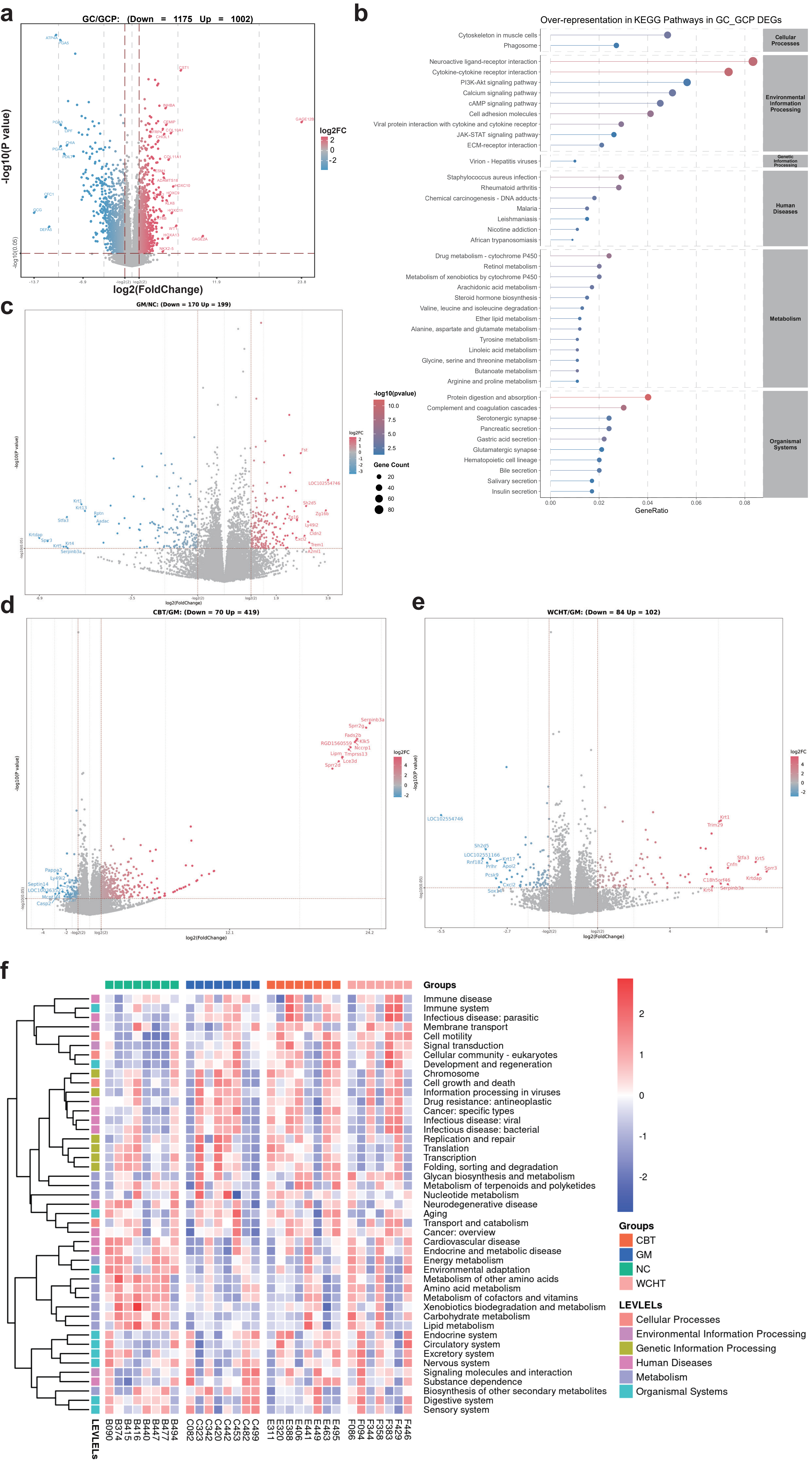

Supplementary Fig. 4 Metatranscriptomic profiling of in-house gastric cancer clinical specimens and rat gastritis intervention models. a, Volcano plot illustrating differential gene expression between gastric cancer tissues (GC) and matched adjacent non-cancerous tissues (GCP). Red dots denote genes upregulated in GC (1175 genes), blue dots represent genes downregulated in GC (1002 genes), with non-gray coloration indicating statistically significant differentially expressed genes (|log2FC|<2 or p>0.05). b, Lollipop chart of KEGG pathway enrichment analysis for GC/GCP differential genes. The top 10 significantly enriched pathways (ranked by GeneRatio) in each KEGG level1. c-e, Volcano plot comparing gene expression profiles between experimental groups in rat intervention studies. In the figure comparing A and B, red coloration denotes upregulated expression in A relative to B, while blue indicates the inverse relationship. Non-gray elements represent genes without statistically significant differential expression (|Fold Change| < 2 and p-value > 0.05). f, Heatmap visualization of GSVA-calculated KEGG pathway activities across experimental groups. Color gradient reflects normalized pathway activity scores. GM: Gastritis model; NC: Normal controls; CBT: Clostridium butyricum treatment group; WCHT: Weichanghao treatment group.
